# The role of RHIM in necroptosis

**DOI:** 10.1042/BST20220535

**Published:** 2022-08-30

**Authors:** Theresa Riebeling, Ulrich Kunzendorf, Stefan Krautwald

**Affiliations:** Department of Nephrology and Hypertension, University Hospital Schleswig-Holstein, 24105 Kiel, Germany

**Keywords:** immunogenic cell death, necroptosis, RHIM domain

## Abstract

The RIP homotypic interaction motif (RHIM) is a conserved protein domain that is approximately 18–22 amino acids in length. In humans, four proteins carrying RHIM domains have been identified: receptor-interacting serine/threonine protein kinase (RIPK) 1, RIPK3, Z-DNA-binding protein 1 (ZBP1), and TIR domain-containing adapter-inducing IFN-β (TRIF), which are all major players in necroptosis, a distinct form of regulated cell death. Necroptosis is mostly presumed to be a fail-safe form of cell death, occurring in cells in which apoptosis is compromised. Upon activation, RIPK1, ZBP1, and TRIF each hetero-oligomerize with RIPK3 and induce the assembly of an amyloid-like structure of RIPK3 homo-oligomers. These act as docking stations for the recruitment of the pseudokinase mixed-lineage kinase domain like (MLKL), the pore-forming executioner of necroptosis. As RHIM domain interactions are a vital component of the signaling cascade and can also be involved in apoptosis and pyroptosis activation, it is unsurprising that viral and bacterial pathogens have developed means of disrupting RHIM-mediated signaling to ensure survival. Moreover, as these mechanisms play an essential part of regulated cell death signaling, they have received much attention in recent years. Herein, we present the latest insights into the supramolecular structure of interacting RHIM proteins and their distinct signaling cascades in inflammation and infection. Their uncovering will ultimately contribute to the development of new therapeutic strategies in the regulation of lytic cell death.

## Introduction

**Figure 1.**
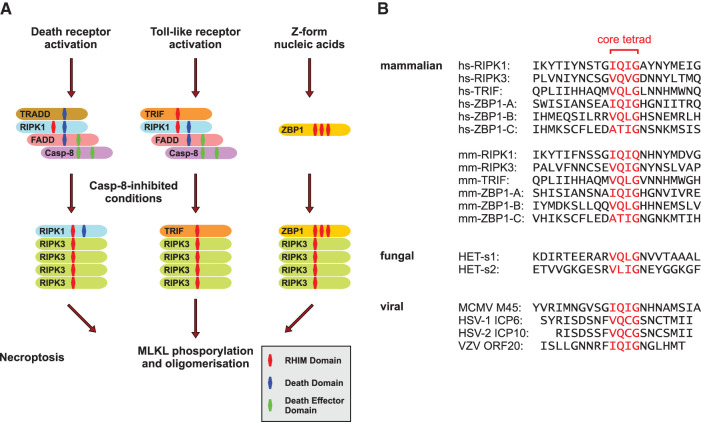
The conserved RHIM domain and its interactions in necroptosis. (**A**) Schematic presentation of the established necroptosis pathways. In response to death receptor signaling, TRADD–RIPK1–FADD–caspase-8 complex assembly is initiated, mediated by death domain and death effector domain interactions. TLR signaling can also directly trigger cell death via assembly of the TRIF–RIPK1–FADD–caspase-8 complex. In caspase-8-inhibited conditions, RIPK1 or TRIF oligomerize with RIPK3 and serve as ‘seeds’ for RIPK3 homo-oligomer formation. Upon sensing intracellular Z-form nucleic acids, e.g. viral RNA, ZBP1 recruits RIPK3 and initiates oligomer formation. The amyloid-like RIPK3 homo-oligomers constitute in each case the platform for MLKL activation, a mandatory step for the execution of necroptotic cell death. (**B**) Alignment of the RHIM amino acid sequence of *Homo sapiens* (hs), *Mus musculus* (mm), the *Podospora anserina* proteins HET-s1 and HET-s2, and the viral RHIM domains of MCMV M45, HSV-1 ICP6, HSV-2 ICP10, and VZV ORF20. The conserved core tetrad is highlighted in red.

RHIM (RIP homotypic interaction motif), an amino acid sequence of approximately 18–22 residues in length [1], is a phylogenetically old motif. Proteins interacting via RHIM domains (Figure 1A) have been described in several metazoan species, with RHIM-like domains found in fungi and even some prokaryotes [2]. The metazoan RHIM domain is structurally and functionally related to the prion-forming domain of the HET-s protein expressed in filamentous fungi [3] (Figure 1B), which oligomerizes and forms fibrils as part of the heterokaryon incompatibility system. Orthologs to mammalian RHIM proteins were discovered in *Drosophila* and *Branchiostoma*, where they are involved in innate immunity signaling pathways [3,4], underlining the conserved nature of this motif.

In mammals, the RHIM domain was originally identified as a short amino acid sequence in the intermediate domain of RIPK1 (receptor-interacting serine/threonine protein kinase 1), and near the RIPK3 C-terminus that was pivotal to protein interactions and the promotion of necroptosis [5,6], a form of regulated necrosis triggered in ischemia-reperfusion injury (IRI), systemic inflammation, autoimmunity, and neurodegenerative diseases, among others [7–16]. Also known as TICAM1, TRIF (TIR domain-containing adapter-inducing interferon (IFN)-β) was the third protein identified to carry a functional RHIM domain and induces a necroptosis-initiating complex with RIPK3 in response to TLR3 (Toll-like receptor 3) and TLR4 signaling [17,18]. The fourth and last protein demonstrated to be able to interact with RIPK3 via a RHIM domain to induce necroptosis is ZBP1, also known as the DNA-dependent activator of interferon (IFN) regulatory factors (DAI) [19–21]. The RHIM domain in these proteins is defined by a core tetrad with the consensus sequence (V/I)-Q-(V/I/L/C)-G, which is essential for intermolecular protein interaction and signal transduction [5,18,19,21–23]. Mutating all four conserved residues to alanine is a common laboratory approach for abolishing RHIM domain signaling and necroptosis without disrupting other functions of the protein [5,19,22,24], whereas mutation of a single valine residue of the RIPK3 core tetrad (mouse RIPK3 V448P corresponding to human RIPK3 V480P) is sufficient to rescue cells *in vitro* from canonical necroptosis [25]. The engagement with RIPK3 via RHIM domain interactions is essential for inducing activation of the effector MLKL (mixed-lineage kinase domain like) [5,19,22,26]. In the following execution phase of necroptosis, MLKL oligomerizes to permeabilize the cell membrane by a mechanism distinct among species that has been investigated in depth in recent years but is not fully understood [27–30]. Subsequently, cell lysis occurs and damage-associated molecular patterns (DAMPs) are released into the extracellular space [31–33]. The importance of RHIM interactions for the promotion of necroptosis signaling is underlined by the finding that while kinase activity of RIPK3 is dispensable for MLKL activation under RIPK3-overexpressed conditions, the RIPK3 RHIM-deficient mutant cannot induce necroptosis despite the fact that it still phosphorylates MLKL. In humans, a core oligomer of at least four RIPK3 protomers interacting via their RHIM domains appears essential to enable MLKL oligomer formation [26].

Identifying the precise order of the necroptosis signaling cascades and the composition of the signaling complexes in response to specific triggers has been the main challenge in past years. Canonically, necroptosis is triggered by death receptor signaling when caspases are inhibited and apoptosis is blocked. Under these circumstances, RIPK3 interacts with activated RIPK1 to initiate necroptosis [6,24], but non-canonical pathways involving RHIM interactions have been discovered. One of these involves the activation of some TLRs. Unlike several other TLRs, both TLR3 and TLR4 can directly initiate necroptotic cell death upon ligand binding via TRIF and do not rely on endocrine or paracrine TNF-dependent mechanisms [17,23]. Most studies have reported that TRIF transitions into a complex with RIPK1, caspase-8, FADD, and cFLIP to initiate apoptosis and interacts with RIPK3 to initiate necroptosis when this pro-apoptotic complex fails [17,34,35]. However, a model for *Yersinia pseudotuberculosis* infection by TLR4 activation under TAK1-inhibited conditions described a RHIM-dependent complex of TRIF, RIPK1, ZBP1, FADD, and caspase-8 termed the TRIFosome, which facilitated a pyroptosis-like form of regulated necrosis [36]. Interestingly, recent studies also uncovered mechanisms in which RIPK3 triggers RIPK1-dependent apoptosis, often as part of the induction of multiple regulated cell death pathways in parallel in response to infection [37–40]. While *in vitro* stimulation using defined cytokines and inhibitors to induce necroptosis has proven valuable to the initial description of RHIM function, we now need to understand these more diverse complexes and their role in inflammation and immunity.

## Host immune evasion by RHIM domain interactions

Viruses infecting mammalian cells often need to dodge detection and triggering the host cell death machinery for long enough to enable efficient replication and spread to other cells [41]. Necroptosis plays a prominent role in the clearance of infected cells and appears to have developed as a backup mechanism to execute cell death when apoptosis fails. However, viral pathogens have co-evolved with the host-regulated cell death machinery and developed mechanisms to ensure cell survival during infection [19,41–43]. In particular, the primate genes encoding RIPK3 and MLKL are in an evolutionary arms race with viral-encoded pathogenicity factors containing functional RHIM domains [34,44]. Specifically, the Betaherpesvirinae, a family of large DNA viruses, utilize an elaborate system to evade several host immune response mechanisms and persist as latent infection [45]. Their mouse-specific member murine cytomegalovirus (MCMV), probably the best studied viral RHIM protein, expresses four different proteins to sequentially block caspase-8-mediated extrinsic apoptosis, RIPK3-mediated necroptosis, and BCL2 family-mediated mitochondrial cell death [42]. By interacting with ZBP1, RIPK1, and RIPK3, MCMV abolishes regulated cell death and TNF-mediated activation of NF-κB and MAPK [46,47]. Infection with an engineered MCMV variant with a mutational disruption of the viral M45 RHIM domain core tetrad led to ZBP1- and RIPK3-dependent but RIPK1-independent necroptosis [20,48], which emphasizes the enormous contribution of this protein to immune evasion and latent infection.

The Alphaherpesvirinae herpes simplex virus (HSV)-1 and HSV-2, both human pathogens, disrupt host cell necroptosis signaling by expressing the RHIM proteins infected cell protein (ICP)6 and ICP10, respectively [49]. As ICP6 simultaneously functions as a suppressor of caspase-8-mediated apoptosis and RIPK3-dependent necroptosis, it is central for establishing HSV infection [49–51]. In the execution of cell death, ICP6 hinders the translocation of the RIPK1–RIPK3–MLKL complex to caveolin-1-associated detergent-resistant membrane (DRM) vesicles, ensuring cell survival in human-derived HT-29 cells [43]. Consistent with that finding, HSV-1 carrying a RHIM-mutated ICP6 failed to evade the cell death of infected human cells and readily induced ZBP1- and RIPK3-dependent necroptosis [52]. Notably, the anti-necroptotic effects mediated by ICP6 are highly host-specific: in mouse cells, it induced RIPK1 and RIPK3 activation and promoted cell death, a finding that can be interpreted as a species barrier contributing to HSV-1 specificity for the natural human host organism [53]. In 2020, another herpesvirus protein with an RHIM domain, ORF20, which is expressed by varicella-zoster virus (VZV) and serves as a decoy for ZBP1, was discovered [37]. RHIM domain mutation inhibited viral spread *in vitro*; however, this mechanism relies on caspase activation rather than canonical necroptosis [37]. Inhibiting RHIM protein-mediated cell death appears to be particularly important in herpesvirus infections and is probably the main contributor to the typical persistent latent infection of these pathogens [45,51,54]. As the reactivation of these infections poses a major challenge in immunocompromised patients [55,56], overcoming pathogen persistence would be a substantial therapeutic advantage: the viral RHIM proteins are promising target structures in this process.

The expression of an interacting RHIM protein is not the only mechanism pathogens utilize to hinder necroptosis in the cells of mammalian host organisms. Many Gram-negative enteric pathogens have evolved bacterial factors that can be delivered to the cytosolic compartment of the infected host's cells via a type III secretion system (T3SS), which manipulates the immunological response by inhibiting inflammatory signaling and immunogenic cell death (reviewed in [57] and [58]). Both enteropathogenic *Escherichia coli* (EPEC) and *Shigella* express a protease delivered by the T3SS that specifically recognizes RHIM sequences in its hosts and thereby cleaves mammalian RHIM proteins [59,60]. The EPEC protease EspL recognizes the RHIM domain conserved core tetrad and cleaves RIPK1, RIPK3, TRIF, ZBP1, and even MCMV M45 [59]. OspD3, the EspL homolog in *Shigella*, also specifically cleaves the RHIM domain of human RIPK1 and RIPK3, extending the survival of infected host cells and thereby protecting the cytosolic replicative niche of the bacterium [60]. The physiological relevance of this pathogenicity factor was experimentally validated in a mouse model of infection with the EPEC-like mouse pathogen *Citrobacter rodentium*. During the resolving stage of the inflammation, EspL-proficient bacteria were more prevalent in the feces of infected animals as compared with an *espL* deletion mutant [59]. This suggested that inhibiting necroptosis additionally promotes bacterial persistence in the gut by limiting the extent of immunogenic cell death. Furthermore, the EspL cleavage site of the RIPK3 RHIM domain in primates was recognized as a rapidly evolving site likely owing to genetic selection [44]. Therefore, it can be assumed that the inhibition of RHIM protein-mediated cell death is a significant virulence factor that contributes to the highly elaborate mechanism utilized by these bacteria to dampen and delay the host immune response [57,59,60].

The depletion of host RIPK3 as a mechanism to enable longer persistence is also utilized by several orthopoxviruses: cowpox virus (CPXV), variola virus, ectromelia virus, and monkeypox virus (currently a worldwide concern); each encodes a viral inducer of RIPK3 degradation (vIRD) [61]. CPXV vIRD binds to the RIPK3 RHIM domain mediated by its N-terminal ankyrin repeats and promotes K48-linked RIPK3 ubiquitination, priming it for proteasomal degradation. In a mouse model of CPXV infection, wild-type virus killed the mice within 5 days while a vIRD-deficient variant was efficiently cleared [61]. However, further investigations will be needed to fully understand how these RIPK3-depleting mechanisms promote pathogenicity and whether they can be targeted in clinical settings to support host defense.

## The RHIM domain assembly — an exceptional structure

It has been established for two decades that the RHIM domain facilitates protein–protein interactions [5], but the ongoing characterization of the nature of these interfaces at the molecular level only began more recently. Surprisingly, it was determined that the motif facilitates the formation of amyloid-like structures defined by fibrils of β-sheet-stabilized protein stacks [62,63]. For the longest time, such amyloid structures in mammals were only associated with pathological settings such as degenerative and prion diseases, but newer evidence showed that amyloids can form as a part of functional signal transduction [64]. Furthermore, several signalosomes involving homotypic interaction with high relevance in cell death and inflammation have been described [62].

The first major issue to understanding RHIM domain function was to determine its molecular structure in the native monomeric proteins. Nuclear magnetic resonance (NMR) studies on the C-terminal domain of human RIPK3 revealed that a sequence spanning 22 amino acids (P448–Q469) forms an S-shaped structure defined by three interacting β-sheets, with the VQVG consensus motif being the central part [65]. Upon homo-oligomerization, this structure assembled into fibrils with the S-shaped core region rotating in each layer, which might be necessary to sterically enable RIPK3 kinase domain positioning and activity [65]. All four human RHIM-expressing proteins have the capacity to self-assemble into such homotypic oligomers with distinct structural characteristics [66,67].

The first stable hetero-amyloid complex identified by structural biology was a heterocomplex of the human-derived RIPK1 and RIPK3 RHIM domains [68]. While the core tetrad was pivotal to proper amyloid formation, several surrounding residues stabilize the defined structure of this complex by their interactions, such as RIPK1 N535 and Q540 with RIPK3 N454 and Q459 [68]. However, Wu et al. showed that the central β-sheet containing the human RIPK3 core tetrad was sufficient to interact with the RIPK1 RHIM motif [65]. It is suggested that the RIPK1/RIPK3 hetero-amyloid core region in humans and mice acts as a ‘seed’ for further RIPK3 homo-amyloid fibrils as amplifiers [1,26,68]. RIPK3-dependent signal transduction appeared to be highly reliant on the correct formation of these RHIM complexes, and substitution mutants of RHIM domain residues substantially impaired downstream signaling [69]. Interestingly, RHIM-deficient human RIPK3 retained the ability to phosphorylate (activate) MLKL but failed to induce MLKL oligomer insertion into the membrane and thereby necroptosis [43]. Again, this underlines the essential role of correct RHIM domain alignment in physiological necroptosis signaling.

As RHIM interactions are highly complex and specifically defined, viral RHIM proteins can efficiently interfere with this system [34]. To understand their mode of action, MCMV M45 protein complexes involving both RIPK1 and RIPK3 have been investigated at the molecular level. In canonical necroptosis signaling, M45 inserts itself into hetero-oligomers with human-derived RIPK3, which results in deformed amyloid-like signaling filaments [69]. When comparing human-derived mixed RIPK3–RIPK1 and RIPK3–M45 hetero-oligomers under conditions allowing fibril assembly, the RIPK3–RIPK1 hetero-oligomer mostly formed single fibrils up to ∼200 nm in length whereas RIPK3–M45 oligomers formed extensive fibrillar networks [66]. HSV-1 ICP6 RHIM interactions with mammalian RHIM proteins have been characterized in more detail: the main interaction partners for ICP6 were identified to be ZBP1 and RIPK3 [52,70] and structural analysis of human-derived recombinant proteins revealed that ICP6–ZBP1 hetero-oligomers were more stable than ZBP1 homo-oligomers, forming longer and thicker fibrils. At the same time, the ICP6–RIPK3 hetero-oligomer was less stable and assembled in shorter fibrils than the RIPK3 homo-oligomer [70]. Studies on these distinct RHIM interactions emphasize the importance of the flanking residues around the core tetrad in shaping the structure of the fibril and defining its activity [66,70,71]. These tightly defined interactions have been positioned as promising targets for synthetic RHIM peptides with the potential to interfere with amyloid assembly to prevent necroptosis in human pathologies. A recent approach to designing synthetic peptides containing the M45 RHIM domain fused to the HIV TAT protein as a delivery system regrettably failed to inhibit necroptosis; on the contrary, the peptides killed the targeted cells by self-assembling into amyloid-like fibrils [72]. However, a 90-amino acid truncated MCMV M45 protein containing the viral RHIM domain associated with RIPK3 more efficiently than RIPK1 and prevented necroptosis *in vitro* [66]. Other than their assembly, the breakdown of higher-order RHIM domain amyloid-like structures are also not fully understood. In 2019, it was reported that autophagy defects led to an accumulation of high-molecular mass structures of TRIF, RIPK1, and RIPK3 — potentially oligomers — and sensitized macrophages to necroptosis [73], but much work remains to be done to understand the underlying process completely.

## ZBP1 — a key mediator in cell fate decision

In recent years, much attention has focused on the role of ZBP1 in the fight against infection due to its role as an intracellular sensor of Z-form nucleic acids, and ZBP1 expression is tightly connected with inflammatory, especially IFN, signaling [74,75]. ZBP1-deficient mice were significantly protected in the setting of systemic inflammatory response syndrome (SIRS) induced by TNF and IFN-γ [75]. As part of influenza A virus (IAV) infection, inflammatory stimuli such as IFNs up-regulate the expression of ZBP1, which is able to interact with RIPK3 after sensing newly synthesized nuclear viral RNA, allowing infected cells to initiate pyroptosis, necroptosis, and apoptosis, an inflammatory pathway for which the term PANoptosis has been defined [76–78]. This multimodal cell death in response to IAV infection involves the activation of regulated cell death pathways via the ZBP1–RIPK3 axis, a complex involving RIPK3 fibril formation to induce necroptosis [77]. Furthermore, caspase-6 associates with this complex, enhancing RIPK3 interaction with ZBP1 and thereby facilitating activation of the NLRP3 inflammasome, a key structure in pyroptosis [79]. The ZBP1–RIPK1–RIPK3 interactions also triggered apoptotic and necroptotic cell death simultaneously in HSV-1-infected glial cells [80], suggesting a global role of ZBP1 for inducing multiple forms of regulated cell death. As IAV infection demonstrates several similarities with severe acute respiratory syndrome coronavirus 2 (SARS-CoV-2) infection in terms of cytokine signaling and cell death, the involvement of ZBP1 in COVID-19 is currently being considered [76,81]. These novel findings highlight the importance of RHIM-mediated signaling as part of inflammatory signaling during infectious diseases.

As part of inflammation, particularly sterile inflammation, necroptosis is a progressive process for which it is imperative to identify inhibitors that limit its pathological extent [7,8,11,82]. In cancer, however, targeted activation of immunogenic regulated cell death supported classical tumor therapy. As ZBP1 promoted necroptosis in response to the sensing of endogenous double-stranded RNA in RIPK1-deficient or caspase-8/FADD-inhibited conditions [83], ZBP1 activation presents opportunities for exploitation in this context. A recent study successfully explored a means of using a Z-DNA formation-triggering small molecule in a cancer model to sensitize tumor cells to ZBP1 activation [39]. Therein, the authors were able to induce necroptosis and apoptosis *in vitro* and re-sensitize tumor cells to treatment with checkpoint inhibitors *in vivo* [39]. In line with these observations, defects in the function of the RNA-editing enzyme adenosine deaminase acting on RNA 1 (ADAR1) induces ZBP1 activation via the accumulation of endogenous double-stranded RNA which, led to pathological cell death and autoinflammation [39,84–86]. Other newly discovered non-canonical activators of RIPK3-mediated cell death are heat stress and osmotic stress [87,88]. In this context, the ZBP1 RHIM-A domain was identified as essential to ZBP1 oligomerization and association with RIPK3 in response to heat shock [87]. However, osmotic stress induced by excess extracellular NaCl or sucrose could also trigger rapid RIPK3-dependent MLKL activation and lytic cell death in human and murine cells but required only an active RIPK3 kinase domain and did not involve RHIM domain interactions [88].

All these novel findings on the role of ZBP1 as a key player in innate immunity present promising opportunities to interfere with inflammatory cell death to improve treatment options in infectious diseases and cancer and suggest the ZBP1 RHIM domains (Figure 1B) as promising targets to mediate cellular processes in a variety of pathologies.

## Conclusions

In this article, the novel insights presented surrounding the RHIM domain, its amyloid-like structures, and its manifold interactions with pathogens all corroborated the importance of this short amino acid sequence to mammalian immunity. In addition, future efforts will aid the discovery of how this highly conserved and specific domain can be exploited as a target structure to therapeutically interfere with immunogenic cell death signaling in necroptosis-associated morbidities and improve the therapeutic options against viral and bacterial pathogens in sterile inflammatory diseases.

## Perspectives

- RHIM-mediated signaling by RIPK1, TRIF, ZBP1, and RIPK3 culminating in necroptosis is a key pathway utilized by cells in inflammation and infection settings. As the RHIM structure is highly conserved and exclusive to regulated cell death signal transduction, it presents a promising target structure for regulating interference.
- The structures of distinct RHIM domains are currently characterized in-depth at the molecular level. Understanding the exact features of these structures will aid the design of RHIM-mediating drugs for therapeutic interventions.
- The nucleic acid sensor ZBP1 is emerging as a promiscuous regulator of cell death in response to virus infection. We are more than aware of the importance of the latter, especially since the outbreak of the infectious disease COVID-19. In particular, ZBP1 signaling could be a target for therapeutic exploitation in infectious diseases in the near future.

## Abbreviations

CPXV: cowpox virus
DAMPs: damage-associated molecular patterns
HSV: herpes simplex virus
IAV: influenza A virus
ICP: infected cell protein
MCMV: murine cytomegalovirus
MLKL: mixed-lineage kinase domain-like
RHIM: RIP homotypic interaction motif
RIPK: receptor-interacting serine/threonine protein kinase
T3SS: type III secretion system
TLR3: Toll-like receptor 3
TRIF: TIR domain-containing adapter-inducing IFN-β
vIRD: viral inducer of RIPK3 degradation
ZBP1: Z-DNA-binding protein 1
